# POPPeT: a New Method to Predict the Protection Factor of Backbone Amide Hydrogens

**DOI:** 10.1007/s13361-018-2068-x

**Published:** 2018-10-18

**Authors:** Jürgen Claesen, Argyris Politis

**Affiliations:** 1 I-BioStat, Hasselt University, Hasselt, Belgium; 2 Department of Chemistry, King’s College London, 7 Trinity Street, London, SE1 1DB UK

**Keywords:** HDX-MS, Protein structure, Protection factor

## Abstract

Hydrogen exchange (HX) has become an important tool to monitor protein structure and dynamics. The interpretation of HX data with respect to protein structure requires understanding of the factors that influence exchange. Simulated protein structures can be validated by comparing experimental deuteration profiles with the profiles derived from the modeled protein structure. To do this, we propose here a new method, POPPeT, for protection factor prediction based on protein motions that enable HX. By comparing POPPeT with two existing methods, the phenomenological approximation and COREX, we show enhanced predictability measured at both protection factor and deuteration level. This method can be subsequently used by modeling strategies for protein structure prediction.

**Electronic supplementary material:**

The online version of this article (10.1007/s13361-018-2068-x) contains supplementary material, which is available to authorized users.

**Graphical Abstract:**
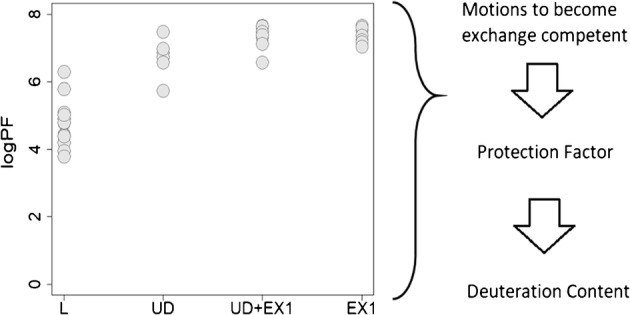
ᅟ

## Introduction

Hydrogen exchange (HX) monitors the exchange of backbone amide hydrogens, providing information about protein structure and dynamics. To interpret the shift in mass and the changes in the isotope distribution with respect to structural properties of a protein, a better understanding of the hydrogen exchange mechanism is needed. Based on the pioneering work of Linderstrøm-Lang [1], a two-state kinetic model that describes HX was proposed [2, 3]:

$$ \mathrm{N}-{\mathrm{H}}_{\mathrm{close}\mathrm{d}}\underset{{\mathrm{k}}_{\mathrm{close}}}{\overset{{\mathrm{k}}_{\mathrm{open}}}{\rightleftharpoons }}\;\mathrm{N}-{\mathrm{H}}_{\mathrm{open}}\;\overset{{\mathrm{k}}_{\mathrm{int}}}{\to}\;\mathrm{N}-{\mathrm{D}}_{\mathrm{open}}\underset{{\mathrm{k}}_{\mathrm{open}}}{\overset{{\mathrm{k}}_{\mathrm{close}}}{\rightleftharpoons }}\mathrm{N}-{\mathrm{D}}_{\mathrm{close}\mathrm{d}} $$

The exchange rate, *k*_ex_, is a function of the intrinsic exchange rate of an unstructured protein, *k*_int_, and of the opening and closing rate constants, *k*_open_ and *k*_close_, or the equilibrium constant, *K*_open_ = *k*_open_/*k*_close_:

1 $$ {k}_{ex}=\frac{k_{open}\times {k}_{int}}{k_{close}+{k}_{open}+{k}_{int}}=\frac{K_{open}}{\left(1+{K}_{open}\right){k}_{int}} $$

The equilibrium constant, *K*_open_, can be considered to be the inverse of the protection factor (PF):

2 $$ \mathrm{PF}\approx 1/{K}_{\mathrm{open}}={k}_{\mathrm{int}}/{k}_{\mathrm{ex}}. $$

In case of the EX2 kinetic exchange regime, the exchange reaction is much slower than the refolding. As a consequence, the unfolding has to happen several times before exchange can take place. The exchange rate:

3 $$ {k}_{ex}=\frac{k_{open}}{k_{close}}\times {k}_{int}={K}_{open}\times {k}_{int} $$

For EX1 kinetics, the exchange takes place during one unfolding/refolding event. As a result, the overall exchange rate is equal to *k*_open_.

Inferring features linked to protein structure from HX data requires an understanding of the factors that exert influence on the HX mechanism. A number of mechanistic models have been proposed throughout the years. Linderstrøm-Lang put forward the idea that slowly exchanging *H* atoms are taking part in hydrogen bonding. These bonds should be, temporarily, broken in order to allow exchange. Solvent-accessibility [4, 5] and solvent-penetration models [6–9] describe an alternative procedure. These models state, respectively, that hydrogens located at the surface exchange at rates close to the intrinsic rates, while amide *H* atoms located in the (hydrophobic) core of the protein exchange slowly. Exchange of the latter requires penetration of the deuterium source in the protein. Other factors that complement the solvent accessibility and solvent penetration models such as acidity and polarizability have also been suggested [10, 11].

Nowadays, it is commonly accepted that the occurrence of *H* bonds is the main determinant of hydrogen exchange [12, 13]. It has been shown that structure-related features such as packing density, or burial, have a limited effect on the HX rates [13–15]. Other factors such as hydrogen bond length and electrostatics have little or no influence on the exchange rates [13–15].

Next to the mechanistic models that try to give insight in the HX mechanism, various predictive models that associate protection or exchange rates with protein structure features have been introduced [16–28]. Even though these methods use different structural and dynamical determinants, and apply different strategies, all models report almost identical accuracy. For each method, small differences between the predicted and measured protection factors are reported. Here, we illustrate what effect these differences have on the deuterium uptake. We also propose a new algorithm for protection factor prediction, namely, *p*rotection fact*o*r *p*rediction based on *p*rotein mo*t*ions (*POPPeT*). We demonstrate accuracy and applicability of POPPeT by comparing it with two existing methods, the phenomenological approximation and COREX, on two proteins, Staphylococcal nuclease A, and equine cytochrome c.

## Methodology

### Determining the Protection Factor

The PF quantifies the degree of reduction in the exchange rate of a backbone amide hydrogen, compared to the intrinsic exchange rate, due to the protein structure. As such, the PF is a function of the protein structure-related features that impede HX. Methods which focus on the prediction of PFs [19–28] can be clustered in two groups: the first group directly associates the protection factor with structure-related features, while the second group indirectly models the protection factor due to its link with the difference in free energy between folded and unfolded states, Δ*G*_ex, *i*_:

4 $$ \Delta {G}_{\mathrm{ex},i}=-\mathrm{RTln}\ {K}_{\mathrm{open},i}=\mathrm{RTln}\ \mathrm{P}{\mathrm{F}}_i $$

We discuss here two commonly applied methods to estimate the protection factor, i.e., the phenomenological approximation [19] and COREX [18]. These methods belong, respectively, to the first and the second group of PF estimation methods.

#### Phenomenological Approximation

According to Vendruscolo and colleagues [21, 22], the protection factor of an amide hydrogen of residue *i* is a function of the number of hydrogen bonds, ${N}_i^H$, and burial, i.e., the number of non-hydrogen atoms within a 6.5 Å distance of the amide nitrogen, ${N}_i^C$:

5 $$ \ln\ {\mathrm{PF}}_i={\beta}_H\times {N}_i^H+{\beta}_C\times {N}_i^C $$

The coefficients *β*_*H*_ and *β*_*C*_ are estimated, when considering backbone atoms, and when considering backbone and side-chain atoms, based on a set of native state simulations for, respectively six, and seven proteins. The reported values are *β*_*C*_ = 0.35 and *β*_*H*_ = 2.0 when considering all atoms [22], and *β*_*C*_ = 1.0 and *β*_*H*_ = 5.0 when considering backbone atoms only [21].

#### COREX

COREX [18] estimates the protection factor of a residue *i* based on the work of Hilser and Freire [16] and Hilser [29]. It generates an ensemble of partially unfolded microstates. The probability of a microstate *s* is calculated as follows:

6 $$ \Pr\ \left(\mathrm{state}\ s\right)=\frac{\exp\ \left(-\varDelta {G}_s/ RT\right)}{\sum_{i=0}^N\exp\ \left(-\varDelta {G}_s/ RT\right)} $$

with Δ*G*_*s*_, the Gibbs free energy, a function of the accessible surface area, and the conformational entropy.

The protection factor of residue *i* is then defined as the ratio of the sum of the probabilities of the microstates in which residue *i* is folded and not exposed to the solvent to the sum of the probabilities of the microstates in which residue *i* is unfolded and solvent accessible:

7 $$ \mathrm{P}{\mathrm{F}}_i=\frac{\sum_{s=1}^{N_i^{\mathrm{folded}}}\Pr \left(\mathrm{state}\ s\right)-\Pr (i)}{\sum_{s=1}^{N_i^{\mathrm{unfolded}}}\Pr \left(\mathrm{state}\ s\right)-\Pr (i)} $$

where *Pr* (*i*) is the sum of the probabilities of the microstates where residue *i* is solvent accessible in its native state, or becomes solvent accessible due to partially unfolding of other residues.

### Linking the Protection Factor with Deuterium Content

The outcomes of protein structure modeling techniques can be validated with HDX-MS data. In these cases, the PFs are calculated based on the proposed protein structures. These PFs are used to calculate the expected deuterium content of a protein at residue level:

8 $$ {D}_i^{\mathrm{time}}=1-\exp\ \left(-{k}_{ex,i}\times \mathrm{time}\right)=1-\exp\ \left\{\left(-{k}_{\mathit{\operatorname{int}},i}/{\mathrm{PF}}_i\right)\times \mathrm{time}\right\} $$

Summing ${D}_i^{\mathrm{time}}$ over *n* contiguous residues gives the expected deuterium content at peptide or protein level. As the exchange of the first residue cannot be recorded due to very fast back exchange, the following equation is used to calculate the theoretical deuteration level for peptides or proteins:

9 $$ {D}^{\mathrm{time}}=\sum \limits_{i=2}^n\left({D}_i^{\mathrm{time}}\right)=\sum \limits_{i=2}^n1-\exp\ \left\{\left(-{k}_{\mathit{\operatorname{int}},i}/{\mathrm{PF}}_i\right)\times \mathrm{time}\right\} $$

By comparing the theoretical peptide deuteration levels with the measured levels, the proposed protein structures with closely matching deuteration profiles are validated and/or selected. Obviously, the accuracy of the predicted protection factors has an effect on this process. However, it remains unclear to which extent the accuracy of the predicted PFs influences the theoretical peptide deuteration levels. Therefore, in addition to the *traditional* methods to determine the PF accuracy, i.e., looking at the difference between the measured and predicted protection factors and the Pearson correlation coefficient [30], we also looked at the difference between the theoretical and measured deuteration levels. Note that additional experimental factors, such as back exchange, can influence the measured deuterium levels and should be accounted for when comparing theoretical deuteration levels with measured levels.

We used Staphylococcal nuclease A (SNase) [31] to calculate the deuterium content of 12 peptides (Table S1) at 16 different time points, ranging from 30 s to 16 days. The intrinsic rate of each residue was calculated with the formulas proposed by Bai et al. [32]. We used the experimentally determined protection factors, as well as the protection factors predicted by the phenomenological approximation, and by COREX as reported by Skinner et al. [14] (Table S3).

### POPPeT, an Alternative Approach to Determine the Protection Factor

According to Skinner et al. [14], the prediction of HX has to be based on the protein motions that generate exchange competent amide hydrogens, i.e., local fluctuations and (global) unfolding reactions. It is possible to determine experimentally if an amide hydrogen becomes exchange competent due to sizeable unfolding or by local fluctuations [33–39]. We introduce a new approach to predict the protection factors of a protein, POPPeT. It is based on information about the protein motions that generate exchange competent backbone amide hydrogens. In the Supplementary Material, we show that there is a statistically significant association between HX-enabling protein motions and logPFs (see Table S4). The information about the HX-enabling protein motions is complemented with a set of structural features including secondary structure elements and hydrogen bonding information (Table 1). It also takes into account other factors such as the number of non-hydrogen atoms in its vicinity (*burial*). These complementary variables are added to clarify part of the variability present in the logPFs that cannot be explained by the considered protein motions.

**Table 1:** Considered structural features. The secondary structure elements are split into three different groups. Hydrogen bonding and protein motions that enable HX are divided in four categories

	Hydrogen bonding	Secondary structure	Protein motions
	Information	Elements	
cat. 1	no	no	L
cat. 2	Hbond with H_2_O	helix	UD
cat. 3	Hbond with main-chain O	*β*-sheet	UD + EX1
cat. 4	Hbond with side-chain O	/	EX1

Information on the secondary structure elements is divided in three categories, i.e., “no,” “helix,” or “*β*-sheet” (Table 1). The category “helix” contains all *H* atoms that are located on residues that form an *α*-helix or a 3/10-helix. Hydrogens from the category “*β*-sheet” are part of amino acids that form a *β*-strand or *β*-bridge. The other backbone hydrogens are grouped in the “no” category. The information about the secondary structure elements is taken from the RCSB protein databank [40].

The exchangeable hydrogens are also grouped into four categories related to hydrogen bonding, i.e., “no,” “Hbond with H_2_O,” “Hbond with main-chain oxygen,” and “Hbond with side-chain oxygen” (Table 1). The hydrogen bonding status is calculated with Chimera [41].

Similar to hydrogen bonding, the protein motions that enable HX are also divided into four categories, i.e., local (“L”), unfolding (“UD”), unfolding and EX1 (“UD + EX1”), and EX1 (“EX1”), as reported by [14, 15]. The category “L” groups the amide hydrogens that get exchange competent through local fluctuations. The other amide hydrogens become exchangeable due to unfolding. We divided them into three subcategories, based on the experimental procedure used to detect unfolding: the addition of denaturant (“UD”), increasing the pH level (“EX1”), and the combination of both (“UD + EX1”). The amide hydrogens that are part of the last two categories exchange with an EX1 mechanism at elevated pH.

In order to predict the logPF based on the selected structural features, and other factors such as burial, we have fitted a log-linear model of the following form:

10 $$ {\displaystyle \begin{array}{l}\mathrm{logPF}={\beta}_0+{\beta}_1\times \mathrm{UD}+{\beta}_2\times \mathrm{EX}1\\ {}\kern2.24em +{\beta}_3\times \left(\mathrm{UD}+\mathrm{EX}1\right)+{\beta}_4\times \mathrm{helix}+{\beta}_5\times \beta -\mathrm{sheet}\\ {}\kern2.24em +{\beta}_6\times \mathrm{burial}+{\beta}_7\times \mathrm{Hbond}\ \mathrm{with}\ {\mathrm{H}}_2\mathrm{O}\\ {}\kern2.24em +{\beta}_8\times \mathrm{Hbond}\ \mathrm{with}\ \mathrm{main}-\mathrm{chain}\ \mathrm{O}\\ {}\kern2.24em +{\beta}_9\times \mathrm{Hbond}\ \mathrm{with}\ \mathrm{side}-\mathrm{chain}\ \mathrm{O}+\varepsilon \end{array}} $$

where *ε* is the residual error, and $\varepsilon \sim \mathcal{N}\left(0,{\sigma}^2\right)$. In the resulting model, only statistically significant parameters are retained (*p* value < 0.05).

This log-linear model directly associates the logPF with structure-related features. As a result, it belongs to the same group of methods as the phenomenological approximation. It differs from the phenomenological approximation as it contains additional information such as information on exchange-enabling motions, and secondary structure features.

## Results

### Accuracy of the Predicted Protection Factors

Existing methods for protection-factor-prediction reported relatively small differences between the predicted and the measured logPFs of exchangeable hydrogens and/or high correlation between them. However, a small difference on the logPF scale does not necessarily imply a small difference on the PF scale. For example, a difference of 2 between the measured and observed logPFs is a much larger difference at the PF scale, i.e., 10^2^. As a consequence, small differences in the logPF scale can have severe effects on the deuteration level, which is a function of the PF (see Eqs. (8) and (9)). To our knowledge, the effect of these differences on the deuteration level has not been studied. We assessed the accuracy of the PFs of amide hydrogens of SNase estimated with the phenomenological approximation and COREX [14, 15].

**Figure 1:**
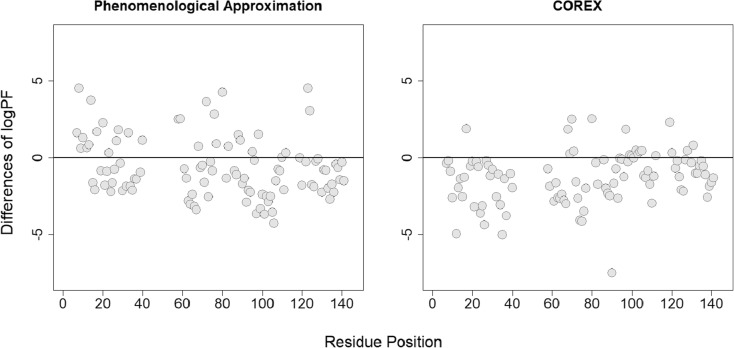
Differences between the predicted and measured logPFs of SNase plotted against the residue positions

We found that for the phenomenological approximation and COREX, only a limited number of protection factors are accurately estimated (Fig. 1). In particular, COREX systematically underestimates the measured logPF, as previously reported in [28]. A potential reason for this systematic underestimation could be the number of hydrogens that are exposed in a microstate, e.g., a smaller number of unfolded residues may increase the PF [16, 29].

**Figure 2:**
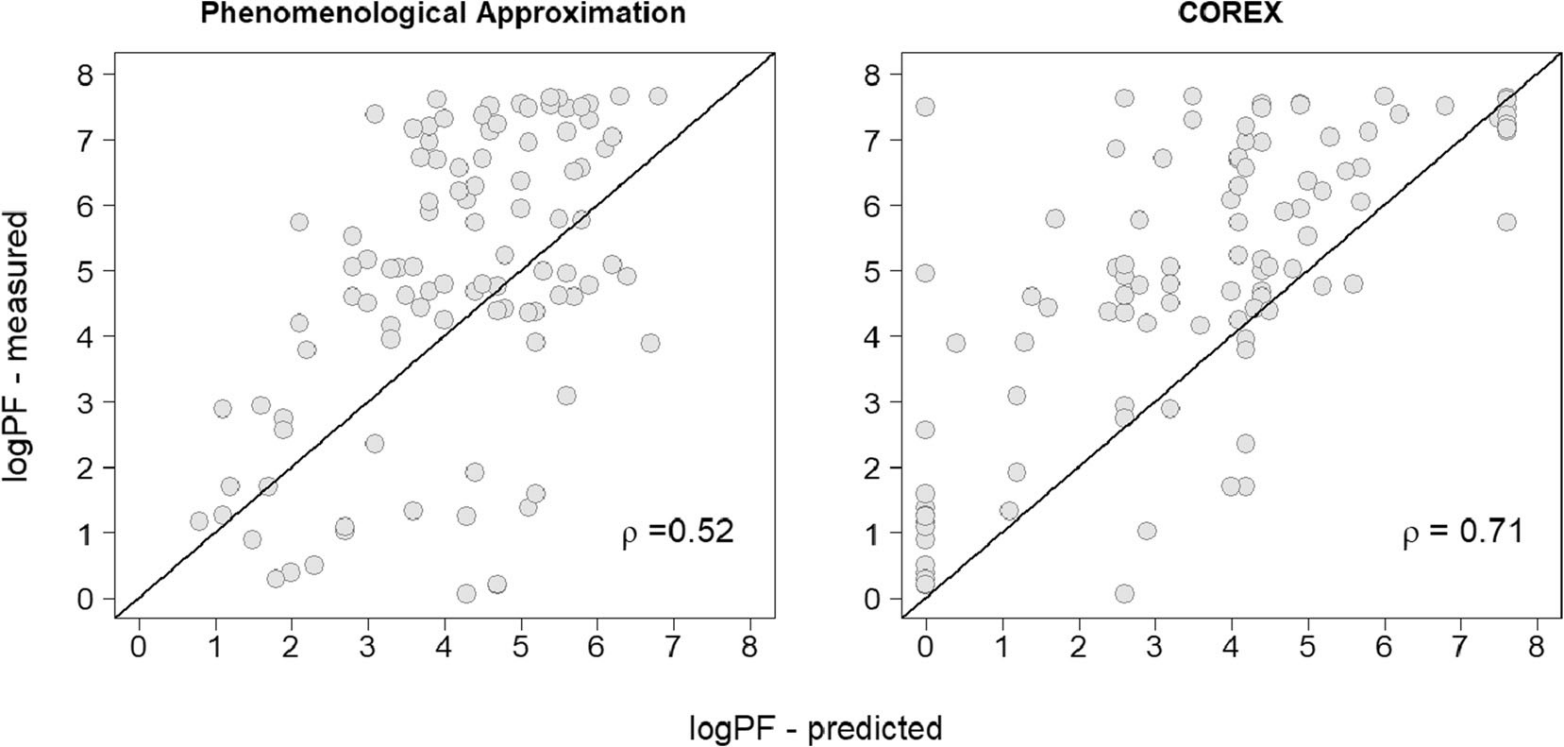
The correlation between the predicted and measured protein factors of SNase. The diagonal line indicates a perfect correlation, i.e., *ρ* = 1.00

The correlation between the predicted and measured protection factors is moderate, i.e., 0.52 and 0.71 for the phenomenological approximation and COREX, respectively (Fig. 2). Based on the observed differences and the moderate correlation, one can expect that there will be discrepancies between the deuterium uptake profiles of SNase peptides, calculated with the measured PFs and the predicted PFs of COREX and the phenomenological approximation.

To quantify the magnitude of these differences in terms of deuteration, we calculated the deuterium content of 12 peptides using the predicted and measured logPFs. The deuteration level of these peptides are calculated, for 16 time points, based on the predicted protection factors of COREX (blue line), the phenomenological approximation (red line), and the measured protection factors (black line) (Fig. S1).

**Figure 3:**
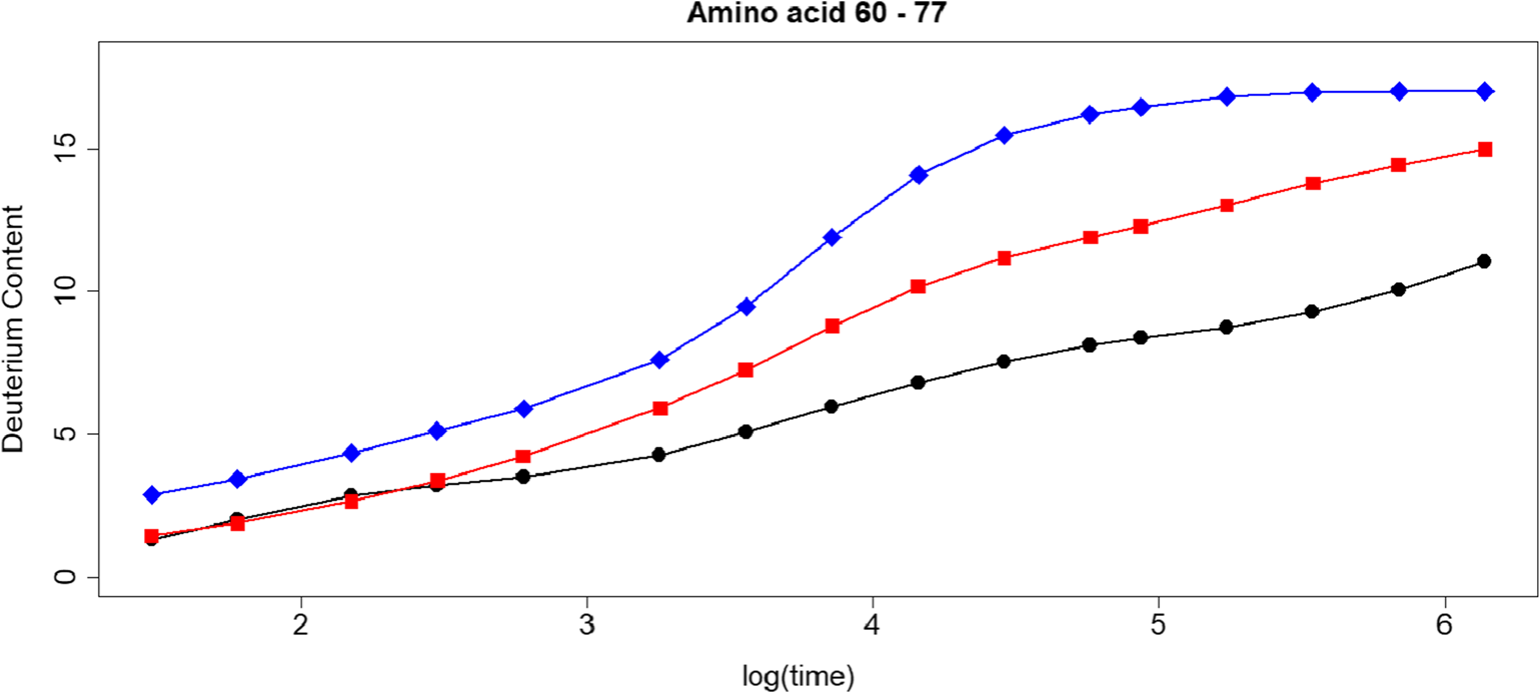
Deuteration profile of SNase peptide 7. The black line is the deuteration level calculated with the measured PFs (∘), the red line with PFs of the phenomenological approximation (□), and the blue line with the PFs of COREX (⋄)

For COREX, the deuteration level of each peptide, except for peptide 10, is consistently higher than the level calculated with the measured PFs. This is in line with the findings of Fig. 1. The differences between the deuteration levels based on the protection factors estimated with the phenomenological approximation and the measured PFs are generally smaller than with COREX, but remain substantial (Fig. S2). For instance, for peptide 7 (Fig. 3), these differences range between − 0.13 (after 60 s of exposure to D) and 4.49 (after 4 days of exchange). In case of COREX, the differences in deuteration vary between 1.50 and 8.12 (see also Table S3). Similar differences can also be seen for the other peptides (Fig. S1). Based on these outcomes, it is clear that when selecting modeled protein structures based on the accordance between the experimental and calculated deuteration profile, one should keep the accuracy of the predicted protection factors in mind.

### POPPeT

#### The Model

For 43 amide hydrogens of SNase, information about their HX-enabling protein motions is available [14]. We randomly split these exchangeable hydrogens in a training set of 30 *H* atoms, and a test set of 13 *H* atoms (Table S5). The training set is used to train the model, i.e., to determine the significant parameters and their effect. The resulting model, based on Eq. (10), has the following form:

11 $$ {\displaystyle \begin{array}{l}\mathrm{logPF}={\beta}_0+{\beta}_1\times \mathrm{UD}+{\beta}_2\times \mathrm{EX}1\\ {}\kern2.24em +{\beta}_3\times \left(\mathrm{UD}+\mathrm{EX}1\right)+{\beta}_4\times \mathrm{helix}+{\beta}_5\times \beta -\mathrm{sheet}\\ {}\kern2.24em +{\beta}_6\times \mathrm{burial}\end{array}} $$

This model differs from the phenomenological approximation as it contains information about the HX-enabling protein motions, taken from [14, 15], and the secondary structure elements next to burial. Hydrogen bonding has no significant effect, probably due to the fact that 27 of the 30 hydrogens form a hydrogen bond with an oxygen from the main chain.

If a hydrogen is part of a helix or a *β*-sheet, its logPF increases, in comparison to a hydrogen atom located in an unstructured part of the protein, with, respectively, 0.60 or 0.36 (Table 2). The PF gets substantially bigger when a hydrogen atom needs to undergo unfolding in order to become exchangeable. For instance, when the unfolding motion is experimentally determined at elevated denaturant levels (UD), the logPF raises with 1.63 (Table 2). Burial also has a significant effect on the logPF. For an exchangeable *H* atom bound to an amide nitrogen which has 30 non-hydrogen atoms within a distance of 6.5 Å, the logPF increases with 30 × 0.04371≈ 1.31 (Table 2). It is worth noting that for the 30 hydrogen atoms in the training set, the average value for burial equals 59.10.

**Table 2:** Parameter estimates for POPPeT

		Estimate	Std. error	*p* value
Intercept	*β*_0_	2.19940	0.59566	0.00120
UD	*β*_1_	1.63431	0.22449	2.08e-7
EX1	*β*_2_	2.12078	0.19786	2.03e-10
UD + EX1	*β*_3_	2.15429	0.18957	6.50e-11
helix	*β*_4_	0.59534	0.19881	0.00647
*β*-sheet	*β*_5_	0.35710	0.18679	0.06844
burial	*β*_6_	0.04371	0.01164	0.00103

The defined categories for the secondary structure elements can be further divided into subcategories. For instance, the category “helix” can be split into “*α*-helix” and “3/10-helix.” However, we found that the adjusted coefficient of determination [42], ${R}_a^2$, of the model with three categories for the secondary structure elements (Table 2) is neglectable smaller (0.04) than the ${R}_a^2$ value of model (11).

#### Accuracy of POPPeT

**Figure 4:**
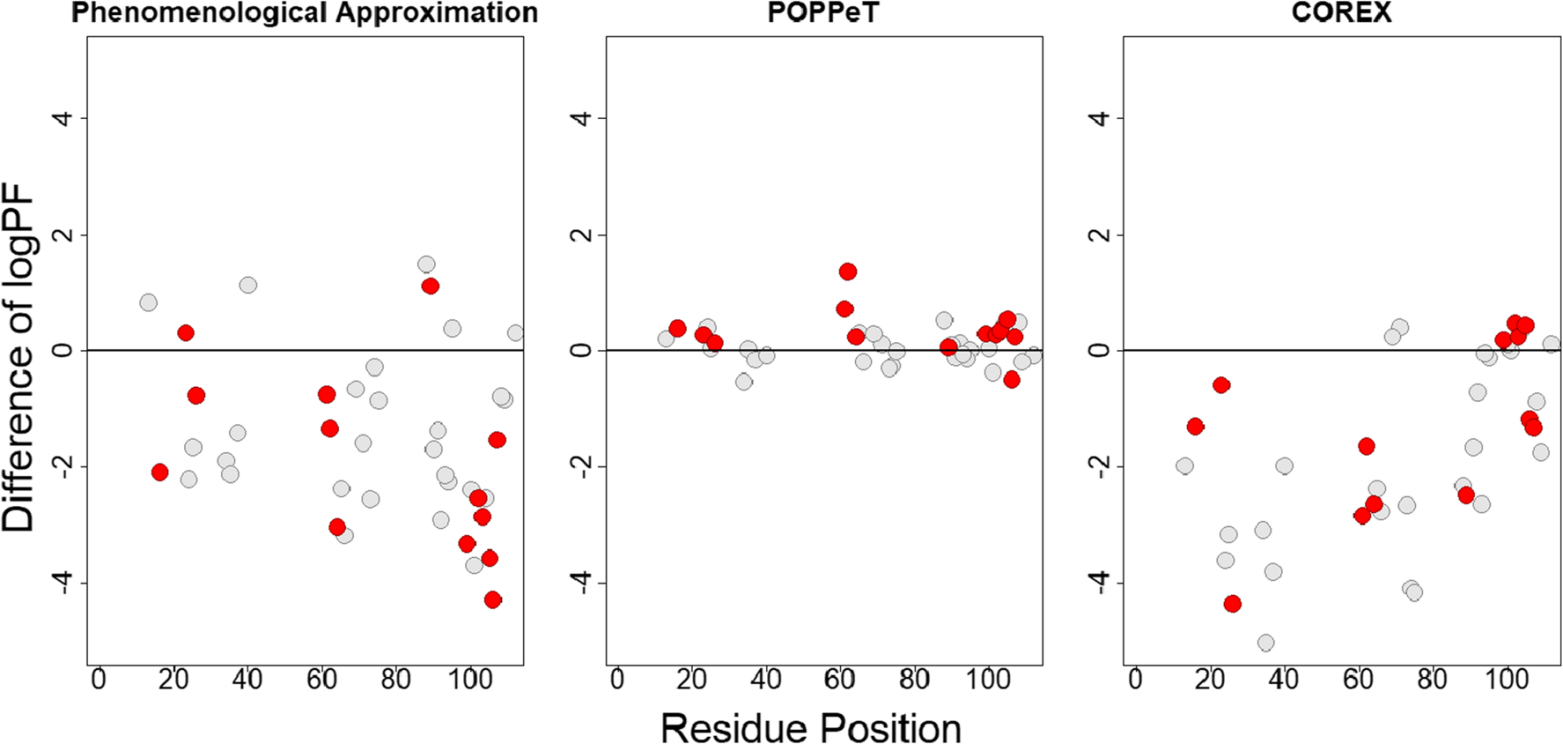
Difference between the predicted and measured logPFs of SNase for the phenomenological approximation (left), POPPeT (center), and COREX (right). The red points are the logPFs from the test set; the gray points are from the training set

**Figure 5:**
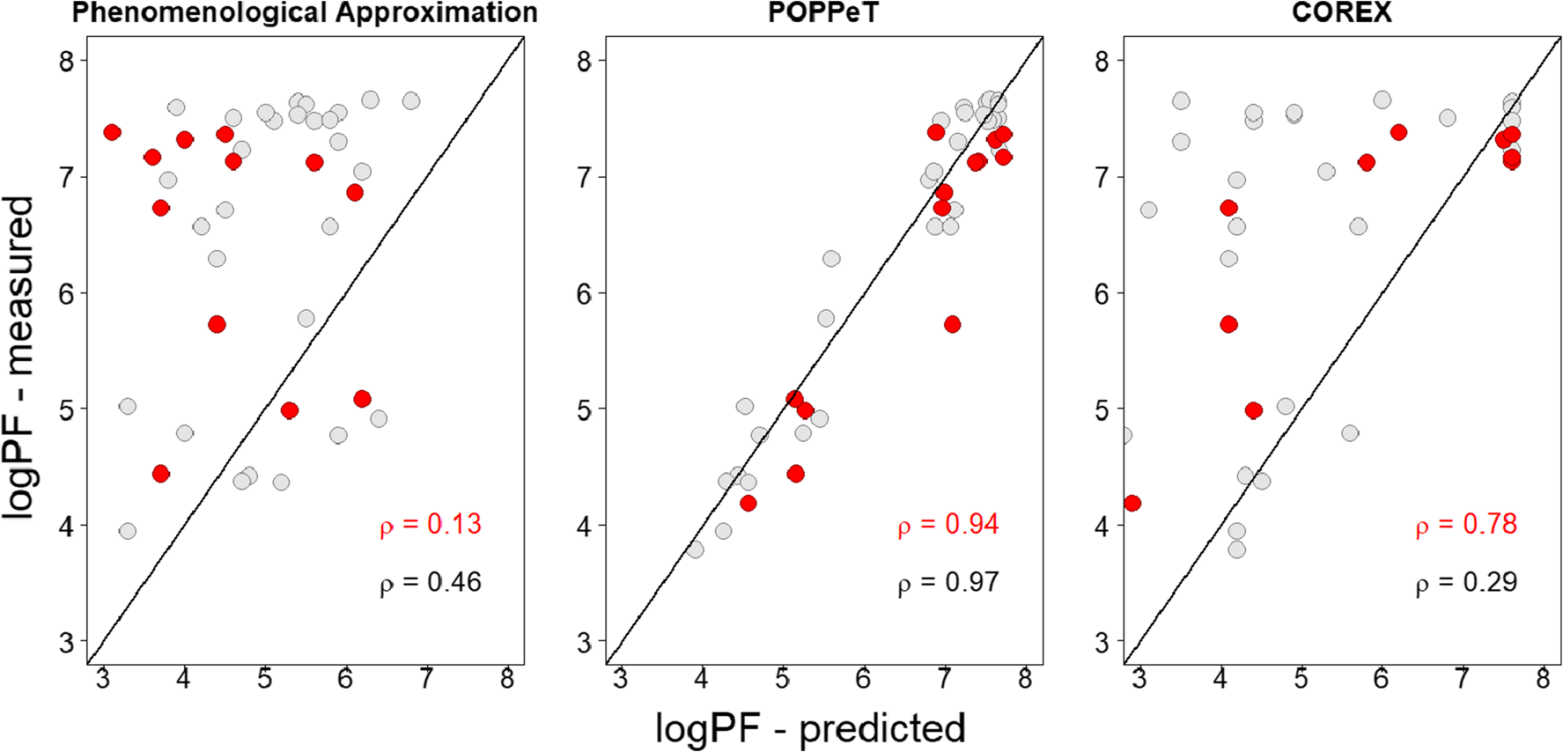
Correlation between the predicted and measured logPFs of SNase. The red points are the logPFs from the test set; the gray points are from the training set

We found that POPPeT accurately predicts the logPFs of the exchangeable SNase hydrogen atoms from the training set (Figs. 4 and 5, in gray). As the training set has been used to estimate the coefficients of the model (11), one should not adhere too much importance to the high accuracy of the training set. For the test set hydrogens of SNase (Table S5), the accuracy and the correlation between the predicted and measured logPFs is high (*ρ* = 0.94) (Figs. 4 and 5, in red). The maximum difference between the measured and predicted logPF is 1.36, which is one third of the absolute maximum differences of the phenomenological approximation (4.28) and COREX (4.36). The average difference between the measured and predicted logPFs is, in case of POPPeT, approximately 4 to 5 times smaller than the averaged differences of the PFs estimated with the phenomenological approximation and with COREX, i.e., 0.41, 2.11, and 1.51, respectively.

**Figure 6:**
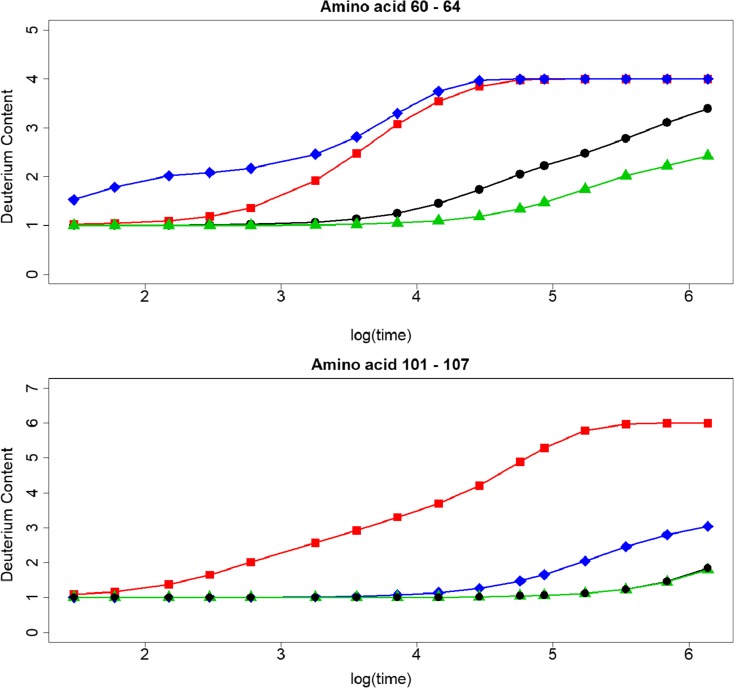
Deuteration profiles of two SNase peptides. The black line is the deuteration level calculated with the measured protection factors (○), the red line with the phenomenological approximation (□), the blue line with COREX (◊), and the green line with POPPeT (△)

Next, we tested the effect of the observed differences between the predicted and measured logPF at the deuteration level. Out of the test set of 13 amino acid SNase residues, we generated two peptides, with amino acid residues from 60 to 64, and from 101 to 107. Amino acids 63 and 104 are not part of the test set. For these two residues, we assumed that the PF of their backbone amide hydrogens was equal to 1. For the first peptide, the deuterium level calculated with the predicted PFs of POPPeT is lower than the deuterium content based on the measured PFs (Fig. 6, top). The maximum difference between these two deuteration levels is 0.96. The maximum difference between the deuteration profile of COREX and the profile based on the measured PFs is much higher, i.e., 2.30. A similar difference could be found for the phenomenological approximation (2.11). For the second peptide (Fig. 6, bottom), there is almost no difference between the deuteration profile calculated with POPPeT and with the measured protection factors. The maximum difference is 0.04. In contrast to this, the maximum difference between the deuterium content calculated with the predicted PFs of the phenomenological approximation and the deuterium content based on the measured PFs equals 4.74. The difference in deuteration between the values derived from COREX and the measured PFs lies in between these two extremes, i.e., a maximum difference of 1.35 is found at the 8-day exposure time point.

We also tested the performance of POPPeT on equine cytochrome c (pdb: 1OCD). We calculated for 34 exchangeable hydrogens of this protein the logPFs with POPPeT and with the phenomenological approximation. For this protein, we have not compared POPPeT with COREX, as results of COREX for oxidized cytochrome c are not publicly available. For POPPeT, we needed information about the exchange-enabling protein motions. However, this information is not readily available. Based on the dependency of Δ*G*_*ex*_ on the concentration of added denaturant, as described by [34], we categorized the amide *H* atoms into three out of the four considered categories of protein motions (Table S6). Hydrogens with no or little change in *taG*_*ex*_ with increasing levels of denaturant are considered to exchange through local fluctuations. As a consequence, we classified them as “L.” Amide *H* atoms requiring partial unfolding to enable hydrogen exchange show a non-linear dependency between Δ*G*_*ex*_ and the denaturant concentration. These hydrogens are grouped into the “UD” category. Lastly, hydrogens with a strong linear dependency between Δ*G*_*ex*_ and the denaturant levels have been categorized as “UD + EX1” as they require global protein unfolding in order to become exchange competent.

**Figure 7:**
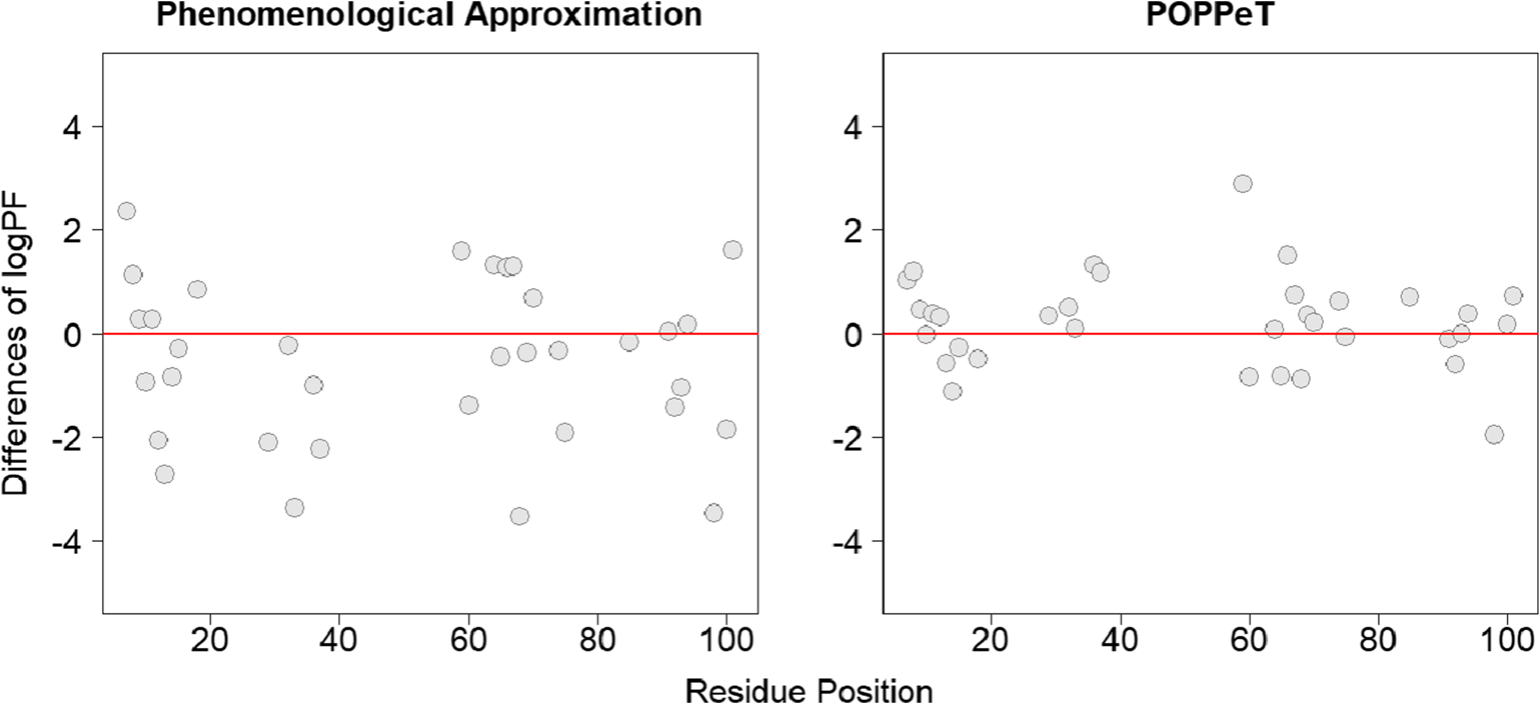
Difference between the predicted and measured logPFs of oxidized cytochrome c for the phenomenological approximation (left) and POPPeT (right)

**Figure 8:**
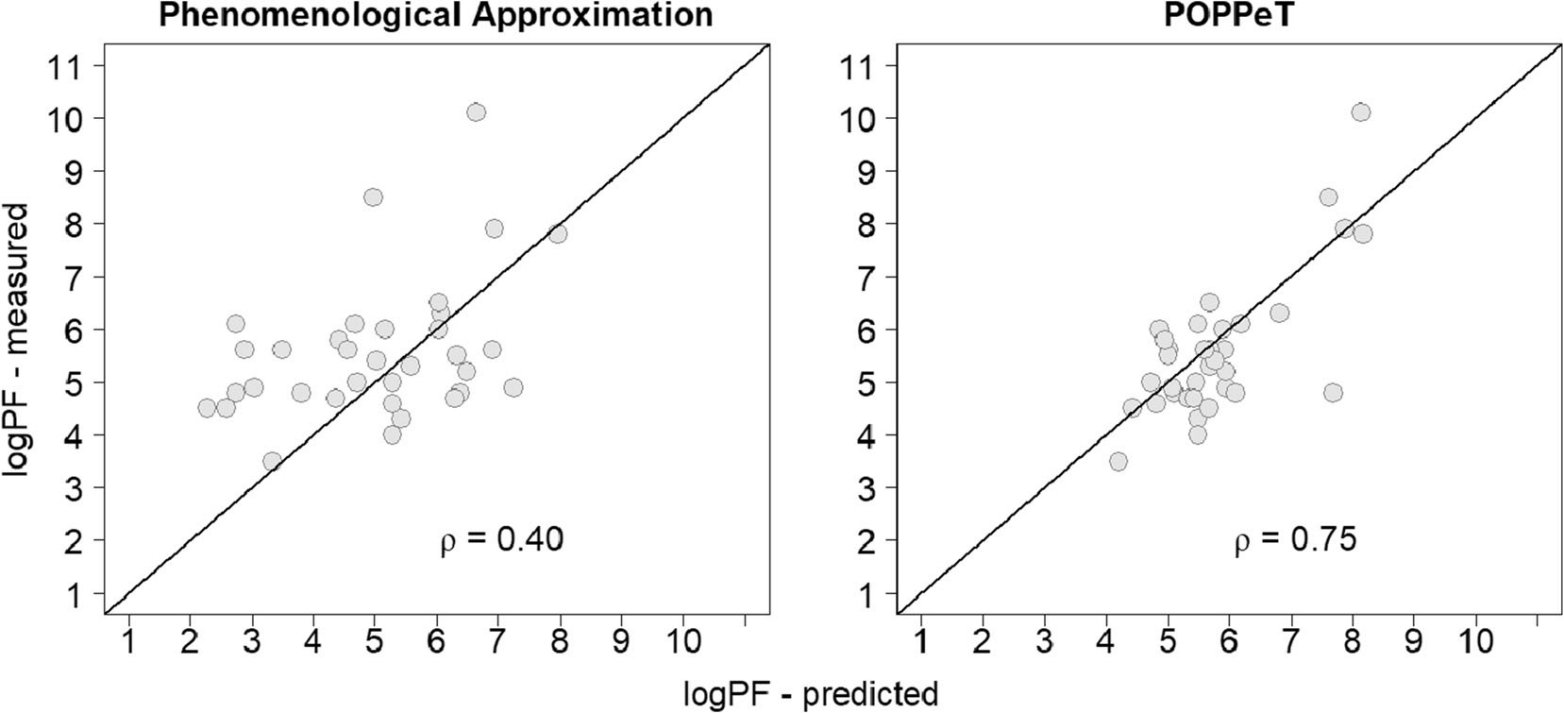
Correlation between the predicted and measured logPFs of oxidized cytochrome c. The correlation between the predicted and measured logPFs is moderately high for POPPeT (right; 0.75), while it is low for the phenomenological approximation (left; 0.40)

Similarly to SNase, the accuracy and the correlation between the predicted and measured logPFs is higher for POPPeT than is the case for the phenomenological approximation (Figs. 7 and 8). The correlation between the measured and predicted PFs is moderately high for POPPeT (*ρ* = 0.75), and low for the phenomenological equation (*ρ* = 0.40). The maximum absolute difference between the predicted and measured PFs is 2.88 for POPPeT and 3.53 for the phenomenological approximation. The average difference between the predicted and measured PFs is approximately two times smaller for POPPeT than for the phenomenological approximation, i.e., 0.68 and 1.31, respectively.

**Figure 9:**
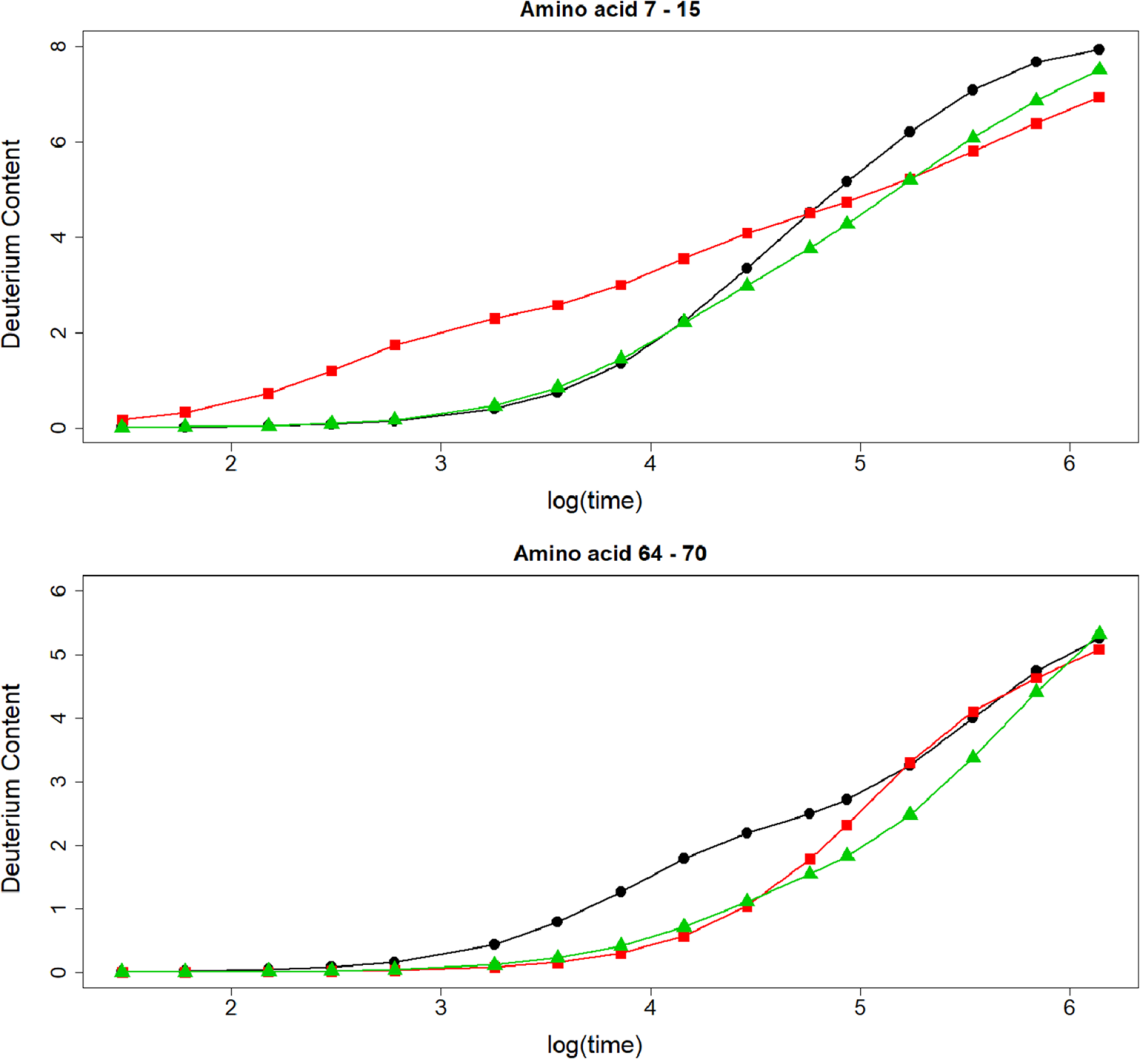
Deuteration profiles of two oxidized cytochrome c peptides. The black line is the deuteration level calculated with the measured protection factors (○), the red line with the phenomenological approximation (□), and the green line with POPPeT (△)

We also tested the effect of the observed differences in logPFs at the deuteration level with two peptides (Fig. 9), from residue 7 to 15, and from residue 64 to 70. We calculated, as before, for each peptide, the deuterium level based on the measured and predicted logPFs at 16 different time points ranging from 30 s to 16 days. For the first peptide, with amino acid residue 7 to 15, the deuteration profiles calculated with the measured PFs and the predicted PFs of POPPeT closely match until time point 10, i.e., 8 h. From this point, the deuterium content based on the output of POPPeT is at most 1.00 Da lower than the deuteration level (Fig. 9, top). There is little or no resemblance between the deuteration profiles based on the measured PFs and the PFs predicted with the phenomenological approximation. The differences in deuteration range from − 1.27 to 1.90. For the second peptide (Fig. 9, bottom), with residues ranging from 64 to 70, no deuteration profile based on the predicted PFs matches closely with the profile calculated from the measured PFs. For POPPeT and for the phenomenological equation, the PFs are overestimated, resulting in a slower predicted deuterium uptake. The maximum difference for POPPeT is 1.08 and for the phenomenological approximation is 1.22.

## Conclusion

In this paper, we presented a new method to estimate the PF of backbone amide hydrogen atoms. POPPeT predicts the PFs based on protein motions with higher accuracy than the existing phenomenological approximation and COREX. Given the small training set used to develop POPPeT, we would like to point out that POPPeT should not be considered as a full-fledged method for protection factor prediction, but rather as a precursor of a number of approaches that use information about HX-enabling protein motions. The statistically very significant association between the logPF and the protein motions that enable HX indicates that whenever this information is available, it should be included in any PF prediction strategy. Using a larger training set to develop POPPeT or similar methods will most likely lead to different parameter estimates, but the overall trends identified here, i.e., hydrogen atoms which become exchange competent through local fluctuations, have a significantly lower PF than hydrogens which require local or global unfolding and will remain statistically significant and thus important for PF prediction. Additionally, we hope that POPPeT and its results will be an encouragement to the structural biology community to (routinely) perform experiments that assess HX-enabling protein motions, and/or will lead to data-driven, computational methods to predict HX-enabling protein motions.

Additionally, we showed that small differences and/or high correlation between the predicted and measured PFs do not necessarily imply small differences in the deuteration level of peptides. A common approach to assess the outcomes of computational methods that predict protein structure is comparing the measured deuteration levels with the predicted deuteration content. When selecting the best matching protein structure, one should keep in mind that the observed differences in deuteration content are not only the result of differences between the true and the predicted protein structure, but that the predicted protection factors also contribute to the differences in deuteration content.

Overall, we expect that POPPeT or other protection factor predictors which incorporate protein motion information will be applicable to computational methods trying to predict the three-dimensional structure of proteins using restraints derived from HDX-MS.

## Electronic Supplementary Material

ESM 1 (PDF 146 kb)
