# Supplementary material for: POPPeT: a New Method to Predict the Protection Factor of Backbone Amide Hydrogens
Source: J Am Soc Mass Spectrom. 2018 Oct 18;30(1):67–76. doi: 10.1007/s13361-018-2068-x (PMC6318252; doi:10.1007/s13361-018-2068-x)
Supplement: Supplementary file 1 — (PDF 146 kb) [file 13361_2018_2068_MOESM1_ESM.pdf]

Supplementary Material

Table S1: Peptides

	Aa-residues	max. D
1	$6 \rightarrow 10$	4
2	$11 \rightarrow 17$	6
3	$18 \rightarrow 30$	12
4	$31 \rightarrow 37$	6
5	$38 \rightarrow 40$	2
6	$57 \rightarrow 59$	2
7	$60 \rightarrow 77$	17
8	$81 \rightarrow 83$	2
9	$85 \rightarrow 112$	27
10	$118 \rightarrow 120$	2
11	$121 \rightarrow 128$	7
12	$129 \rightarrow 141$	12

Table S2: Staphylococcal nuclease information

Residue	logPF	sec. struct. elem.	Hbond acceptor (res.)	Hbond acceptor (atom)	burial	Phenomenological approx.	COREX
Thr2	-1.10						
Lys5	0.43						
Leu7	0.38	no	/	/	28	2.0	0.0
His8	0.19	no	HOH	O	40	4.7	0.0
Lys9	0.88	β-strand	HOH	O	45	1.5	0.0
Glu10	3.90	β-strand	VAL	O	55	5.2	1.3
Ala12	4.95	β-strand	ILE	O	48	5.6	0.0
Thr13	4.37	β-strand	MET	O	46	5.2	2.4
Leu14	1.37	β-strand	HOH	O	46	5.1	0.0
Ile15	5.04	β-strand	LYS	O	45	3.4	2.5
Lys16	4.19	β-strand	LYS	O	46	2.1	2.9
Ala17	1.01	β-strand	HOH	O	51	2.7	2.9
Asp19	4.16	bend	THR	O	53	3.3	3.6
Gly20	1.33	bend	HOH	O	59	3.6	1.1
Asp21	4.60	bend	/	/	57	2.8	1.4

Residue	logPF	sec. struct. elem.	Hbond acceptor (res.)	Hbond acceptor (atom)	burial	Phenomenological approx.	COREX
Thr22	4.68	β-strand	/	/	63	3.8	4.4
Val23	4.99	β-strand	PHE	O	62	5.3	4.4
Lys24	6.71	β-strand	LYS	O	67	4.5	3.1
Leu25	7.55	β-strand	MET	O	66	5.9	4.4
Met26	6.86	β-strand	THR	O	64	6.1	2.5
Tyr27	4.60	β-strand	GLN	O	67	5.7	4.4
Lys28	0.49	turn	HOH	O	44	2.3	0.0
Gly29	1.17	turn	HOH	O	33	0.8	0.0
Gln30	5.17	β-strand	TYR	O	42	3.0	4.4
Met32	6.96	β-strand	LEU	O	53	5.1	4.4
Thr33	1.09	β-strand	HOH	O	52	2.7	0.0
Phe34	7.48	β-strand	VAL	O	63	5.6	4.4
Arg35	7.62	β-strand	GLY	O	68	5.5	2.6
Leu36	6.37	β-strand	ASP	O	60	5.0	5.0
Leu37	7.30	turn	ALA	O	64	5.9	3.5
Val39	5.95	β-strand	/	/	55	5.0	4.9
Asp40	4.77	β-strand	LYS	O	49	5.9	2.8

Residue	logPF	sec. struct. elem.	Hbond acceptor (res.)	Hbond acceptor (atom)	burial	Phenomenological approx.	COREX
Ala58	1.92	α-helix	TYR	O	60	4.4	1.2
Ser59	3.08	α-helix	GLY	O	58	5.6	1.2
Phe61	4.44	α-helix	GLU	O	54	3.7	1.6
Thr62	5.73	α-helix	ALA	O	61	4.4	4.1
Lys63	6.70	α-helix	SER	O	61	3.9	4.1
Lys64	6.73	α-helix	ALA	O	58	3.7	4.1
Met65	6.57	α-helix	PHE	O	56	4.2	4.2
Val66	6.97	α-helix	THR	O	54	3.8	4.2
Glu67	7.20	α-helix	LYS	O	54	3.8	4.2
Asn68	2.35	no	LYS	O	41	3.1	4.2
Ala69	3.95	no	VAL	O	47	3.3	4.2
Lys70	1.70	bend	ASP	OD1	37	1.2	4.2
Lys71	3.79	no	ASP	OD1	39	2.2	4.2
Ile72	1.58	β-strand	HOH	O	50	5.2	0.0
Glu73	7.55	β-strand	TYR	O	58	5.0	4.9
Val74	5.78	β-strand	GLU	O	68	5.5	1.7
Glu75	7.65	β-strand	TYR	O	68	6.8	3.5

Residue	logPF	sec. struct. elem.	Hbond acceptor (res.)	Hbond acceptor (atom)	burial	Phenomenological approx.	COREX
Phe76	3.88	β-strand	HOH	O	61	6.7	0.4
Asp77	4.61	no	HOH	O	62	5.5	2.6
Gln80	0.06	no	GLN	OE1	35	4.3	2.6
Thr82	2.93	β-bridge	/	/	47	1.6	2.6
Asp83	4.35	no	ARG	O	51	5.1	2.6
Gly86	2.75	bend	ASP	O	40	1.9	2.6
Arg87	4.61	no	ASP	OD1	55	3.5	2.6
Gly88	4.91	β-strand	THR	O	66	6.4	2.6
Leu89	5.08	β-strand	ARG	O	59	6.2	2.6
Ala90	7.49	β-strand	ARG	O	66	5.8	0.0
Tyr91	7.66	β-strand	GLU	O	65	6.3	6.0
Ile92	7.51	β-strand	ASN	OD1	68	4.6	6.8
Tyr93	7.53	β-strand	GLU	O	64	5.4	4.9
Ala94	7.64	β-strand	LYS	O	64	5.4	7.6
Asp95	4.42	turn	LYS	O	51	4.8	4.3
Gly96	1.26	turn	HOH	O	40	1.1	0.0
Lys97	5.74	β-strand	ALA	O	40	2.1	7.6

Residue	logPF	sec. struct. elem.	Hbond acceptor (res.)	Hbond acceptor (atom)	burial	Phenomenological approx.	COREX
Met98	0.28	β-strand	HOH	O	56	1.8	0.0
Val99	7.32	α-helix	ILE	O	61	4.0	7.5
Asn100	7.48	α-helix	/	/	59	5.1	7.6
Glu101	7.60	α-helix	/	/	53	3.9	7.6
Ala102	7.13	α-helix	MET	O	57	4.6	7.6
Leu103	7.36	α-helix	VAL	O	64	4.5	7.6
Val104	7.23	α-helix	ASN	O	63	4.7	7.6
Arg105	7.17	α-helix	GLU	O	64	3.6	7.6
Gln106	7.38	turn	ALA	O	58	3.1	6.2
Gly107	7.12	turn	VAL	O	69	5.6	5.8
Leu108	6.57	bend	LEU	O	62	5.8	5.7
Ala109	7.04	no	/	/	58	6.2	5.3
Lys110	5.77	β-strand	ASP	O	61	5.8	2.8
Val111	5.90	β-strand	GLU	OE2	52	3.8	4.7
Ala112	4.38	no	LEU	O	48	4.7	4.5
Asn119	1.70	no	/	/	63	1.7	4.0
Thr120	2.89	turn	ASP	OD2	53	1.1	3.2

Residue	logPF	sec. struct. elem.	Hbond acceptor (res.)	Hbond acceptor (atom)	burial	Phenomenological approx.	COREX
Glu122	4.68	α-helix	ASN	O	56	4.4	4.0
Gln123	0.20	α-helix	HOH	O	48	4.7	0.0
Leu124	1.25	α-helix	HOH	O	52	4.3	0.0
Leu125	6.08	α-helix	HIS	O	53	4.3	4.0
Arg126	6.29	α-helix	GLU	O	64	4.4	4.1
Lys127	4.24	α-helix	GLN	O	48	4.0	4.1
Ser128	4.76	α-helix	LEU	O	56	4.7	5.2
Ala130	6.05	α-helix	ARG	O	53	3.8	5.7
Gln131	4.79	α-helix	LYS	O	56	4.0	5.6
Ala132	6.52	α-helix	SER	O	75	5.7	5.5
Lys133	6.21	α-helix	GLU	O	69	4.2	5.2
Lys134	5.52	α-helix	ALA	O	57	2.8	5.0
Glu135	5.02	turn	GLN	O	53	3.3	4.8
Lys136	5.06	turn	LYS	O	55	2.8	4.5
Leu137	5.23	no	ALA	O	64	4.8	4.1
Asn138	2.57	3/10-helix	HOH	O	54	1.9	0.0
Ile139	5.05	3/10-helix	GLY	O	58	3.6	3.2

Residue	logPF	sec. struct. elem.	Hbond acceptor (res.)	Hbond acceptor (atom)	burial	Phenomenological approx.	COREX
Trp140	4.80	3/10-helix	LEU	O	54	4.5	3.2
Ser141	4.50	no	ASN	O	41	3.0	3.2
Asn144	0.47						
Ala145	0.09						
Asp146	0.16						
Ser147	-0.22						
Gly148	0.32						
Gln149	-0.35						

Figure S1: Deuteration plots for SNase. The black lines are calculated with the measured protection factors, the red lines with the phenomenological approximation, and the blue lines with COREX.

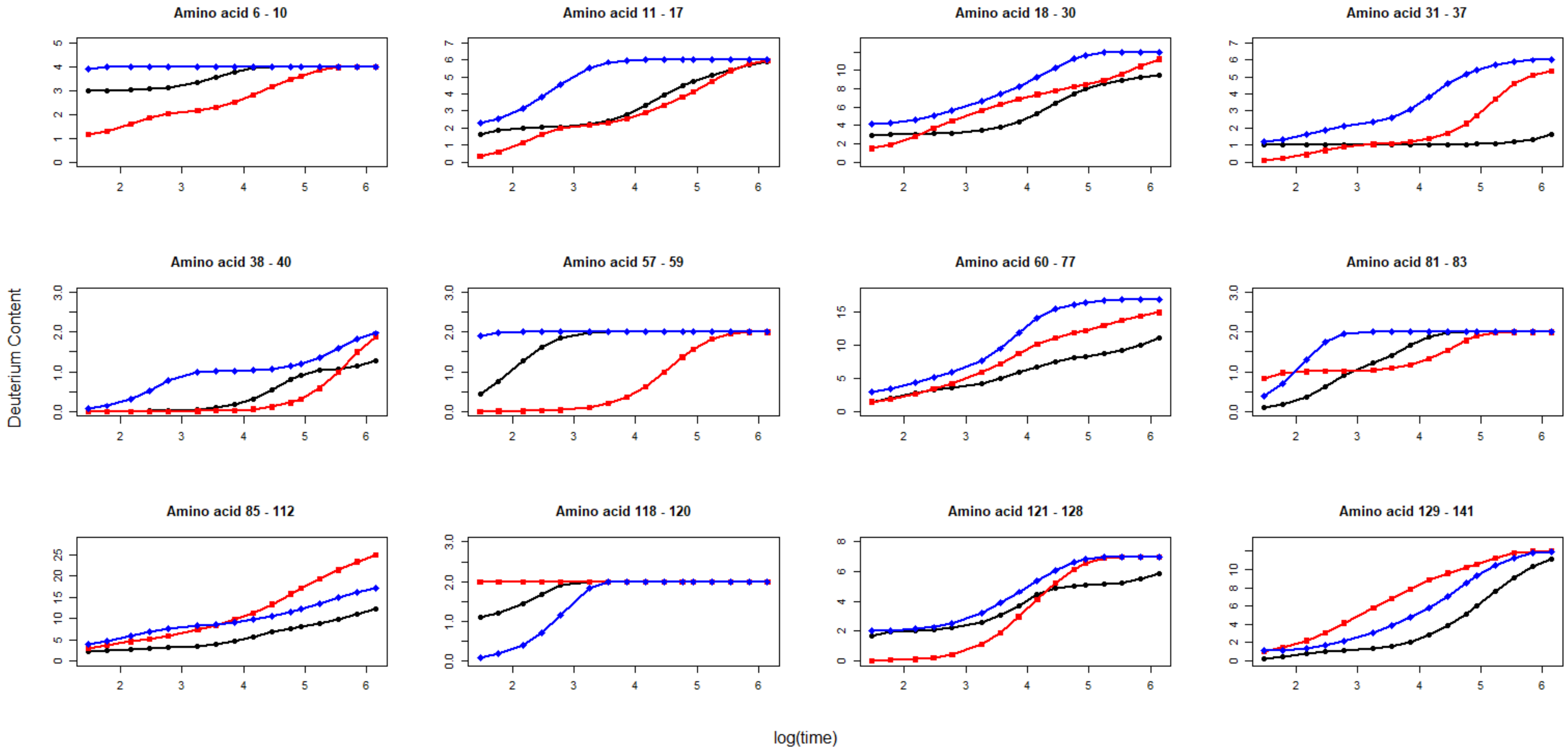

Figure S2: Differences between the measured and calculated deuteration level

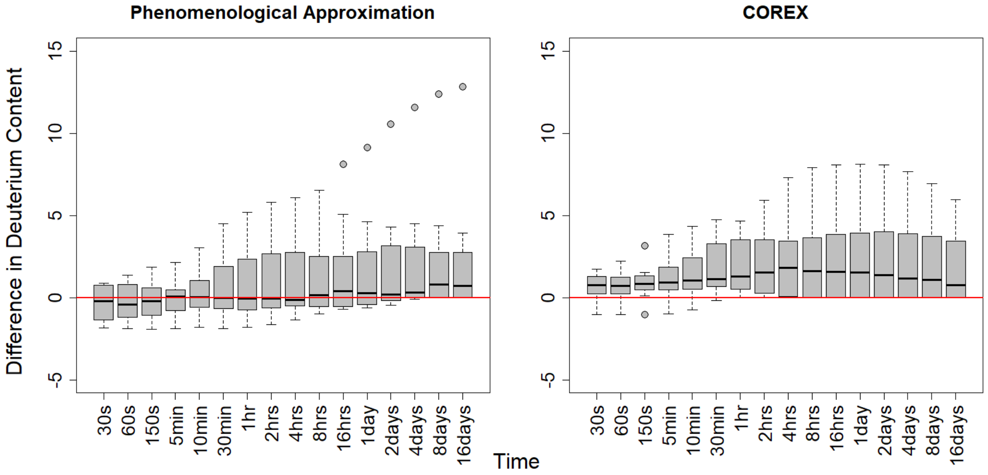

Association between protection factors and protein motions enabling exchange

Knowledge about the protein motions that enable hydrogen exchange is considered to be essential to understand HX behavior and the link between these motions and PFs. As a consequence, we assessed the association between these motions and the protection factors for 43 SNase amide-hydrogens.

A difference between the PFs of these hydrogens that become exchangeable through local fluctuations ('L'), and hydrogens that get exposed due to unfolding ('U') was observed (Figure S1). The average logPF of the first group is 4.74, while it equals 7.21 for the latter.

We further divided the hydrogens that become exchangeable due to unfolding into three subcategories: unfolding due to the addition of denaturant ('UD'), transition of the EX2 to the EX1-mechanism at elevated pH ('EX1'), and the combination of both ('UD+EX1'). PF-differences can also be seen between these three sub-categories (Figure S3, right), especially between 'UD' and 'UD+EX1', and 'UD' and 'EX1'. The average logPFs for

Table S3: Differences between the measured and calculated deuteration level for peptide 7.

	Phenomenological approximation	COREX
30s	0.1416	1.5784
60s	-0.1292	1.4301
150s	-0.2052	1.5020
5min	0.1467	1.8874
10min	0.7279	2.3851
30min	1.6253	3.3211
1hr	2.1749	4.4080
2hrs	2.8330	5.9243
4hrs	3.3984	7.3098
8hrs	3.6436	7.9321
16hrs	3.7732	8.0785
1day	3.9187	8.1232
2days	4.2770	8.0684
4days	4.4950	7.6746
8days	4.3579	6.9326
16days	3.9349	5.9472

these three subcategories are 6.72, 7.37 and 7.38.

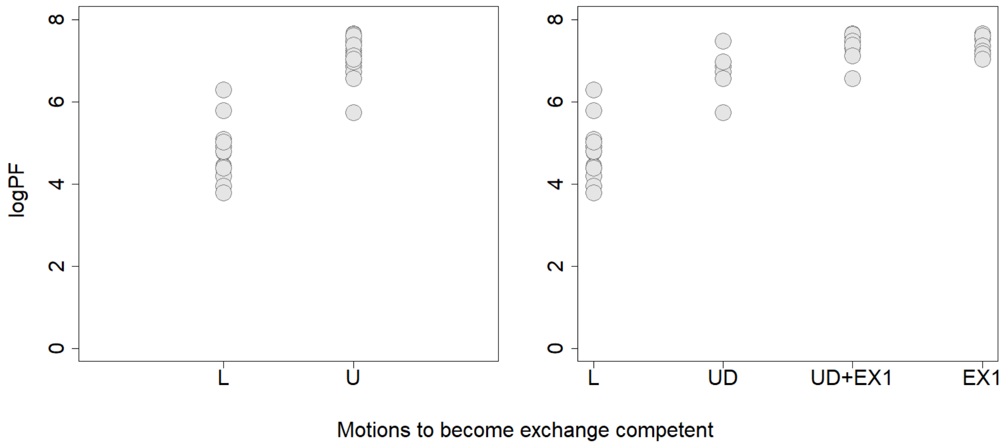

Figure S3: The measured protein factors versus the motions to become exchange competent.

The association between the protection factors and the protein motions that enable HX have been tested with following ANOVA-models:

$$\begin{aligned}\text{logPF} &= \beta_0 + \beta_1 \times \text{U} \\ \text{logPF} &= \beta_0 + \beta_1 \times \text{UD} + \beta_2 \times (\text{UD+EX1}) + \beta_3 \times \text{EX1}\end{aligned}$$

For both models, a significant association between the protein motions and the protection factors was found (F-stat = 214; p-value $<$ 2.2e-16, and F-stat = 87.7; p-value <2.2e-16; see also Table S4). This significant association indicates that knowledge about HX-enabling protein motions can be used to predict the PFs of backbone amide hydrogens, possibly in combination with structural features and other factors.

Table S4: F-statistic and coefficients of the linear models to test the association between protection factor and HX enabling protein motions. The standard errors of the coefficients are between brackets.

	'L' and 'U' (all)	'L', 'U', 'U+EX1', and 'EX1'
F-stat	214 (df 1,41)	87.7 (df 3,39)
p-value	< 2.2e-16	< 2.2e-16
β_0 ('L')	4.7447 (0.1361)	4.7447 (0.1250)
β_1 ('U' - 'L')	2.4678 (0.1687)	1.9768 (0.2216)
β_2 ('U+EX1' - 'L')	/	2.6244 (0.1922)
β_3 ('EX1' - 'L')	/	2.6393 (0.1977)

Table S5: Amino-acid residues of SNase used in the test set

	Residue	Position	logPF
1	Lys	16	4.19
2	Val	23	4.99
3	Met	26	6.86
5	Phe	61	4.44
6	Thr	62	5.73
7	Lys	64	6.73
8	Leu	89	5.08
9	Val	99	7.32
10	Ala	102	7.13
11	Leu	103	7.36
13	Arg	105	7.17
14	Gln	106	7.38
15	Gly	107	7.12

Table S6: Equine oxidized cytochrome c information

	Residue	Position	logPF	Unfolding information [48]	protein motions
1	Lys	7	4.9	local	L
2	Lys	8	4.3	local	L
3	Ile	9	5.0	local	L
4	Phe	10	7.9	partial	UD
5	Val	11	5.3	local	L
6	Gln	12	4.8	local	L
7	Lys	13	5.6	local	L
8	Cys	14	6.0	local	L
9	Ala	15	5.0	local	L
10	His	18	5.5	local	L
11	Gly	29	5.6	partial	UD
12	Leu	32	6.3	partial	UD
13	His	33	6.1	partial	UD
14	Phe	36	4.8	partial	UD
15	Gly	37	4.5	partial	UD
16	Trp	59	4.8	global	UD + EX1
17	Lys	60	5.8	local	L
18	Leu	64	5.6	local	L
19	Met	65	6.5	local	L
20	Glu	66	4.0	local	L
21	Tyr	67	5.2	local	L
22	Leu	68	8.5	global	UD + EX1
23	Glu	69	5.4	local	L
24	Asn	70	4.6	local	L
25	Tyr	74	4.7	local	L
26	Ile	75	4.5	local	L
27	Ile	85	3.5	local	L
28	Arg	91	6.0	local	L
29	Glu	92	6.1	local	L
30	Asp	93	5.6	local	L
31	Leu	94	7.8	global	UD + EX1
32	Leu	98	10.1	global	UD + EX1
33	Lys	100	4.9	local	L
34	Ala	101	4.7	local	L
